# Acid–base sites synergistic catalysis over Mg–Zr–Al mixed metal oxide toward synthesis of diethyl carbonate†

**DOI:** 10.1039/c7ra13629c

**Published:** 2018-01-26

**Authors:** Tingting Yan, Weihan Bing, Ming Xu, Yinwen Li, Yusen Yang, Guoqing Cui, Lan Yang, Min Wei

**Affiliations:** State Key Laboratory of Chemical Resource Engineering, Beijing Advanced Innovation Center for Soft Matter Science and Engineering, Beijing University of Chemical Technology Beijing 100029 P. R. China yanglan@mail.buct.edu.cn weimin@mail.buct.edu.cn

## Abstract

In heterogeneous catalysis processes, development of high-performance acid–base sites synergistic catalysis has drawn increasing attention. In this work, we prepared Mg/Zr/Al mixed metal oxides (denoted as Mg_2_Zr_*x*_Al_1−*x*_–MMO) derived from Mg–Zr–Al layered double hydroxides (LDHs) precursors. Their catalytic performance toward the synthesis of diethyl carbonate (DEC) from urea and ethanol was studied in detail, and the highest catalytic activity was obtained over the Mg_2_Zr_0.53_Al_0.47_MMO catalyst (DEC yield: 37.6%). By establishing correlation between the catalytic performance and Lewis acid–base sites measured by NH_3_-TPD and CO_2_-TPD, it is found that both weak acid site and medium strength base site contribute to the overall yield of DEC, which demonstrates an acid–base synergistic catalysis in this reaction. In addition, *in situ* Fourier transform infrared spectroscopy (*in situ* FTIR) measurements reveal that the Lewis base site activates ethanol to give ethoxide species; while Lewis acid site facilitates the activated adsorption of urea and the intermediate ethyl carbamate (EC). Therefore, this work provides an effective method for the preparation of tunable acid–base catalysts based on LDHs precursor approach, which can be potentially used in cooperative acid–base catalysis reaction.

## Abstract 2

Mg/Zr/Al mixed metal oxides were prepared *via* a facile phase transformation process of hydrotalcite precursors, which showed acid–base sites synergistic catalytic performance toward the synthesis of diethyl carbonate from ethanol and urea.

## Graphical abstract

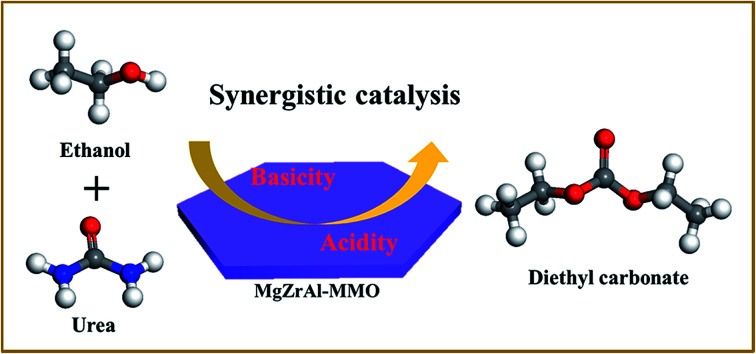

## 1. Introduction

Dialkyl carbonates have attracted widespread interest during the last decades due to their extensive industrial applications.^1^ As an important homologue of the dialkyl carbonate family, diethyl carbonate (DEC) has become increasingly important as a green solvent, a replacement of some toxic substances such as phosgene, dimethyl sulphate and alkyl halide in carbonylation and alkylation reactions, and an electrolyte in lithium ion batteries.^2–4^ Additionally, DEC can be used as an ideal additive for gasoline for its higher oxygen content and favorable fuel/water distribution coefficient with negligible environmental pollution.^5^ Up to now, DEC can be synthetized *via* several routes: phosgenation of ethanol,^6^ oxidative carbonylation of ethanol,^7,8^ transesterification of organic carbonates,^9,10^ and ethanolysis of urea,^11,12^ among which ethanolysis of urea is particularly promising due to cost effectiveness, mild reaction conditions and safe operations. Furthermore, this process involves an indirect utilization of CO_2_: the by-product ammonia can be recycled and further reacts with CO_2_ to produce urea, which is sustainable and friendly process. Zinc oxide (ZnO) catalyst has been commonly used in this reaction with a relatively high yield of DEC, but suffered from dissolution of solid ZnO during the reaction.^13^ Therefore, the exploration of highly efficient, stable catalysts is desirable. Previous studies have shown that both acidity and basicity are crucial to determine the catalytic performance in ethanolysis of urea;^14,15^ however, the cooperation of acid–base sites as well as their respective contribution to catalytic performance are unclear. Thus, how to tune the acid–base properties of catalysts so as to achieve a synergistic catalysis with a high stability and efficiency remains a challenge.

Layered double hydroxides (LDHs)^16,17^ are a class of anionic clay materials with alternating cationic brucite-type layers and charge-balancing anions located in the interlayer region, which can be represented by the formula [M_1−*x*_^2+^M_*x*_^3+^(OH)_2_](A^*n*−^)_*x*/*n*_·*m*H_2_O. In recent years, LDHs materials have attracted an intensive attention as catalysts and catalyst supports in heterogeneous catalysis, owing to their tunability in chemical composition and phase transformation from metal hydroxides to mixed metal oxides (MMOs) upon calcination treatments.^18^ If the chemical composition and element ratio in LDHs host matrix are modulated, the resulting MMOs would possess versatility in concentration and intensity of Lewis acid–base sites. This inspires us to explore the synthesis of LDH precursors and precise control over their structural transformation process, for the purpose of attaining acid–base sites synergistic catalysis toward synthesis of DEC.

In this work, Mg/Zr/Al mixed metal oxides (denoted as Mg_2_Zr_*x*_Al_1−*x*_–MMO) were prepared by a facile phase transformation process from Mg_2_Zr_*x*_Al_1−*x*_–LDH precursors, and their catalytic performance toward DEC synthesis from urea and ethanol was investigated. A tunable acid–base site can be obtained *via* changing the Zr/Al molar ratio in the LDHs precursors. The Mg_2_Zr_0.53_Al_0.47_MMO catalyst demonstrates the best catalytic behavior with a DEC yield of 37.6%, which is among the highest level compared with previous studies. It is found that the synergistic catalysis between weak Lewis acid and medium strength Lewis base site is responsible for the DEC production: medium strength base site facilitates the activated adsorption of ethanol while weak acid site activates urea and intermediate ethyl carbamate (EC, the intermediate production from the reaction of ethanol and urea), which is revealed by *in situ* FTIR measurements.

## 2. Experiment section

### 2.1 Materials

Chemical reagents, including Mg(NO_3_)_2_·6H_2_O, ZrO(NO_3_)_2_·*x*H_2_O, Al(NO_3_)_3_·9H_2_O, NaOH, Na_2_CO_3_, ethanol, urea, and cyclohexanol was purchased and used without further purification. Deionized water was used in all the experimental processes.

### 2.2 Synthesis of MgZrAl–LDH precursors and MgZrAl–MMO catalysts

MgZrAl–LDH precursors with various compositions were synthesized by a co-precipitation method. Typically, Mg(NO_3_)_2_·6H_2_O, ZrO(NO_3_)_2_·*x*H_2_O and Al(NO_3_)_3_·9H_2_O with a Mg^2+^/(Zr^4+^ + Al^3+^) molar ratio of 2 were dissolved in 200 mL of deionized water to give a solution with a total cationic concentration of 0.1 M (solution A). NaOH (2 M) and Na_2_CO_3_ (0.3 M) were dissolved in water (100 mL) to obtain a base solution (solution B). Solution B was added dropwise to solution A at 45 °C with vigorous stirring until the pH value of the slurry reached to 10. The slurry was aged at 75 °C for 12 h, and the obtained precipitate was washed thoroughly and dried in an oven at 60 °C overnight. The resulting sample is labeled as Mg_2_Zr_*x*_Al_1−*x*_–LDH, where *x* is the molar ratio of Zr/(Zr + Al). Subsequently, the Mg_2_Zr_*x*_Al_1−*x*_–LDH precursor was calcined at 550 °C for 4 h in a N_2_ atmosphere at a heating rate of 5 °C min^−1^. The calcination process results in the phase transformation from Mg_2_Zr_*x*_Al_1−*x*_–LDH precursor to MgO–ZrO_2_–Al_2_O_3_ mixed metal oxides (denoted as Mg_2_Zr_*x*_Al_1−*x*_–MMO).

### 2.3 Catalytic evaluation

The catalytic performance was conducted on a stainless steel autoclave (50 mL) equipped with a magnetic stirrer. In a typical method, the catalyst, urea, and ethanol were packed into the reaction reactor. The air in the reaction reactor was replaced by N_2_ three times and then sealed. The reaction was performed at 200 °C for 5 h. The product was analyzed off-line by using gas chromatograph (Shimadzu GC-2014C equipped with a flame ionization detector). The internal standard method was applied for the quantitative analysis with cyclohexanol as internal standard. For recycling tests, the used catalyst was separated from the liquid by centrifugation, washed thoroughly and dried, and then reused for the next reaction cycle.

For the reaction of urea and ethanol, the yield of diethyl carbonate (DEC) or ethyl carbamate (EC) was determined by the formulas:1
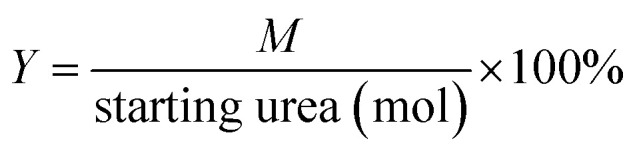
where *M* represents the cumulative mole number of DEC or EC, respectively.

### 2.4 Characterization

Transmission electron microscopy (TEM) and high-resolution TEM (HRTEM) observations were carried out on a JEOL JEM-2100 transmission electron microscope. The morphology of the samples was investigated using a Zeiss Supra 55 scanning electron microscope (SEM) with an accelerating voltage of 20 kV. Powder X-ray diffraction (XRD) patterns were obtained on a Rigaku XRD-6000 diffractometer, using Cu Kα radiation (*λ* = 0.15418 nm) at 40 kV, 30 mA, a scanning rate of 10° min^−1^, and a 2*θ* angle ranging from 3 to 90°. Low-temperature N_2_ adsorption–desorption isotherms of the samples were obtained on a Quantachrome Autosorb-1C-VP analyzer. Prior to N_2_ adsorption, the sample was outgassed at 200 °C overnight. The total specific surface area was evaluated from the multipoint Brunauer–Emmett–Teller (BET) method.

*In situ* Fourier-transformed infrared absorption of pyridine (Py-IR) spectra were obtained using a Nicolet 380 instrument spectrophotometer. The sample (20 mg) was pressed into a self-supporting wafer and installed in an IR cell with CaF_2_ window, activated at 450 °C for 1 h in a flow of N_2_, and then the temperature was cooled down to 150 °C for recording the background spectrum. Subsequently, pyridine was introduced into the cell at 150 °C for 1 h. The spectra were recorded after the sample was purged with flowing N_2_ to remove physisorbed pyridine.

Temperature-programmed desorption (TPD) was performed by using a Micromeritics AutoChem II 2920 apparatus, equipped with a thermal conductivity detector (TCD). Prior to adsorption, the sample (100 mg) was pretreated in a helium flow (50 cm^3^ min^−1^) at 823 K for 1 h (heating rate: 10 °C min^−1^) and then cooled to 50 °C. The sample was exposed to CO_2_ or NH_3_ for 0.5 h, and then was maintained in a He flow for 1 h to remove physisorbed CO_2_ or NH_3_. Afterwards, the temperature was increased linearly at a rate of 10 °C min^−1^ to 600 °C for recording the signal of CO_2_ or NH_3_.

*In situ* Fourier transform infrared spectroscopy (*in situ* FTIR) measurements were carried out using a Nicolet 380 instrument spectrophotometer with 4 cm^−1^ of resolution. For the adsorption study of urea (or EC), 2 mg of urea (or EC) and 20 mg of catalyst were pressed into a self-supporting wafer, and then was packed into the IR cell. In the case of ethanol adsorption, the catalyst (20 mg) was pressed into a self-supporting wafer and installed in the IR cell, followed by the introduction of ethanol vapor. IR spectra were recorded in the temperature range 30–210 °C.

## 3. Results and discussion

### 3.1 Structural and morphological studies on catalysts

**Fig. 1:**
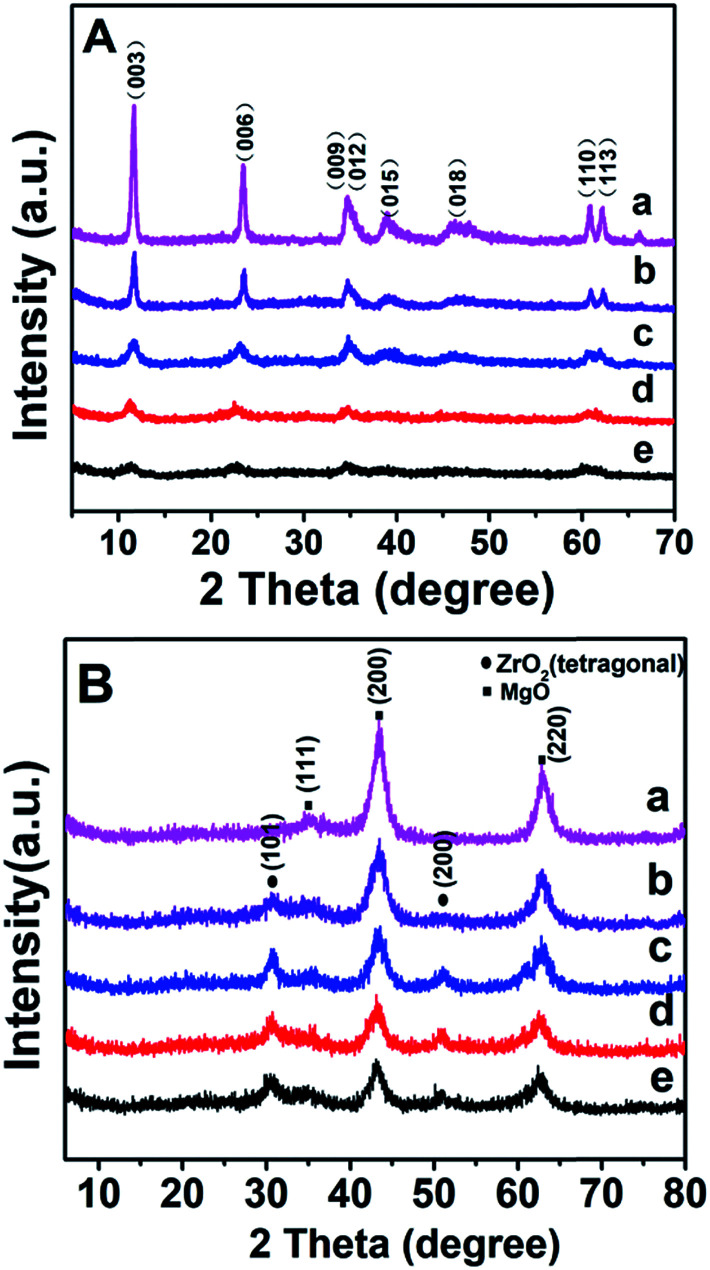
(A) XRD patterns of LDH precursors: (a) Mg_2_Al–LDH, (b) Mg_2_Zr_0.22_Al_0.78_–LDH, (c) Mg_2_Zr_0.33_Al_0.67_–LDH, (d) Mg_2_Zr_0.53_Al_0.47_–LDH, and (e) Mg_2_Zr_0.67_Al_0.33_–LDH. (B) XRD patterns of (a) Mg_2_Al–MMO, (b) Mg_2_Zr_0.22_Al_0.78_–MMO, (c) Mg_2_Zr_0.33_Al_0.67_–MMO, (d) Mg_2_Zr_0.53_Al_0.47_–MMO, and (e) Mg_2_Zr_0.67_Al_0.33_–MMO. Crystalline phase: (●) ZrO_2_, (■) MgO.

Fig. 1A shows the XRD patterns of as-synthesized Mg_2_Zr_*x*_Al_1−*x*_–LDH precursors with various Mg/Zr/Al molar ratios. All the samples exhibit characteristic Bragg reflections at 2*θ* 12° and 24°, which can be indexed to (003) and (006) of an LDH structure (JCPDS no. 35-0964), respectively. However, the crystallinity of Mg_2_Zr_*x*_Al_1−*x*_–LDH decreases gradually with the increase of Zr content, probably owing to the distortion in LDH layers caused by the substitution of Al^3+^ (ionic radius 0.053 nm) by Zr^4+^ (ionic radius 0.072 nm). Calcination of LDH precursors at 550 °C results in the structural transformation from LDHs to mixed metal oxides. XRD patterns of Mg_2_Zr_*x*_Al_1−*x*_–MMO samples (Fig. 1B) give superimposition of two kinds of oxide phases: Bragg reflections at 2*θ* 36°, 43° and 63° are indexed to (111), (200) and (220) of periclase MgO phase (JCPDS no. 45-0946) and those at 2*θ* 30° and 51° indicate the existence of tetragonal ZrO_2_ (t-ZrO_2_) phase (JCPDS no. 42-1164). Interestingly, with the increase of Zr/Mg molar ratio, the peak intensity of MgO phase decreases gradually while that of ZrO_2_ phase enhances progressively. Additionally, no reflection of Al_2_O_3_ is observed, indicating an amorphous phase.

**Fig. 2:**
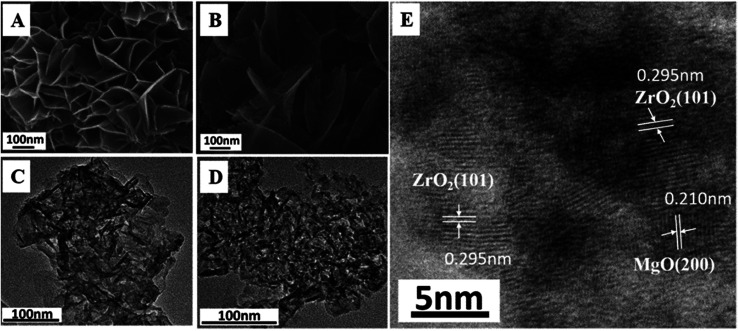
SEM images of (A) Mg_2_Zr_0.53_Al_0.47_–LDH, (B) Mg_2_Zr_0.53_Al_0.47_–MMO. TEM images of (C) Mg_2_Zr_0.53_Al_0.47_–LDH, (D) Mg_2_Zr_0.53_Al_0.47_–MMO. (E) HRTEM image of Mg_2_Zr_0.53_Al_0.47_–MMO.

As a typical sample, the structural transformation from Mg_2_Zr_0.53_Al_0.47_–LDH precursor to Mg_2_Zr_0.53_Al_0.47_–MMO was studied by SEM, TEM and HRTEM measurements (Fig. 2). The Mg_2_Zr_0.53_Al_0.47_–LDH precursor (Fig. 2A and C) shows smooth and uniform thin platelet nanocrystal; Mg_2_Zr_0.53_Al_0.47_–MMO still inherits the original lamellar morphology of LDH precursor (Fig. 2B and D), but with a porous structure resulting from the calcination process. In addition, HRTEM image of Mg_2_Zr_0.53_Al_0.47_–MMO sample (Fig. 2E) exhibits two identified lattice fringe distances of 0.210 nm and 0.295 nm, which are indexed to the (200) plane of MgO and (101) plane of t-ZrO_2_ phase, respectively. This displays a homogeneous dispersion of MgO and t-ZrO_2_ in an amorphous Al_2_O_3_ platelet matrix, in good accordance with the XRD results.

**Fig. 3:**
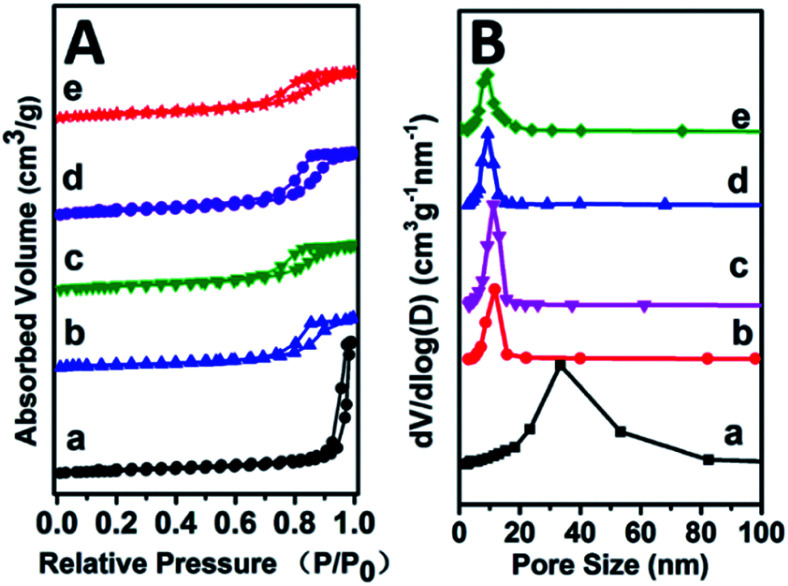
(A) N_2_ adsorption–desorption curves and (B) pore size distributions of: (a) Mg_2_Al–MMO, (b) Mg_2_Zr_0.22_Al_0.78_–MMO, (c) Mg_2_Zr_0.33_Al_0.67_–MMO, (d) Mg_2_Zr_0.53_Al_0.47_–MMO, (e) Mg_2_Zr_0.67_Al_0.33_–MMO.

Specific surface area and pore-size distribution of all these Mg_2_Zr_*x*_Al_1−*x*_–MMO samples were studied by the nitrogen adsorption–desorption isotherms and porosity measurements. All the samples display IV type isotherms with H3 shaped hysteresis loops, which are typical characteristic of mesoporous materials and plate-like particles (Fig. 3). As listed in Table 1, with the increase of Zr content, the surface area shows an increase trend from 113.27 m^2^ g^−1^ to 159.45 m^2^ g^−1^ and then decreases to 143.64 m^2^ g^−1^, with an average pore diameter ranging in 8–10 nm. Noticeably, in comparison with the Mg_2_Al–MMO sample (111.12 m^2^ g^−1^), the Mg_2_Zr_*x*_Al_1−*x*_–MMO samples show higher surface area (113.27–159.45 m^2^ g^−1^), as a result of the introduction of Zr, which would be favorable in heterogeneous catalysis process.

**Table 1:** Structural parameters of various samples

Sample	*S*_BET_ a/m^2^ g^−1^	*D*_BJH_ b/nm	*V*_BJH_ c/cm^3^ g^−1^
Mg_2_Al–MMO	111.12	30.87	1.11
Mg_2_Zr_0.22_Al_0.78_–MMO	113.27	10.28	0.41
Mg_2_Zr_0.33_Al_0.67_–MMO	129.16	9.78	0.57
Mg_2_Zr_0.53_Al_0.47_–MMO	159.45	8.32	0.43
Mg_2_Zr_0.67_Al_0.33_–MMO	143.64	8.21	0.42

a BET surface area.

b BJH desorption average pore diameter.

c BJH desorption cumulative pore volume.

### 3.2 Acid–base properties of catalysts

**Fig. 4:**
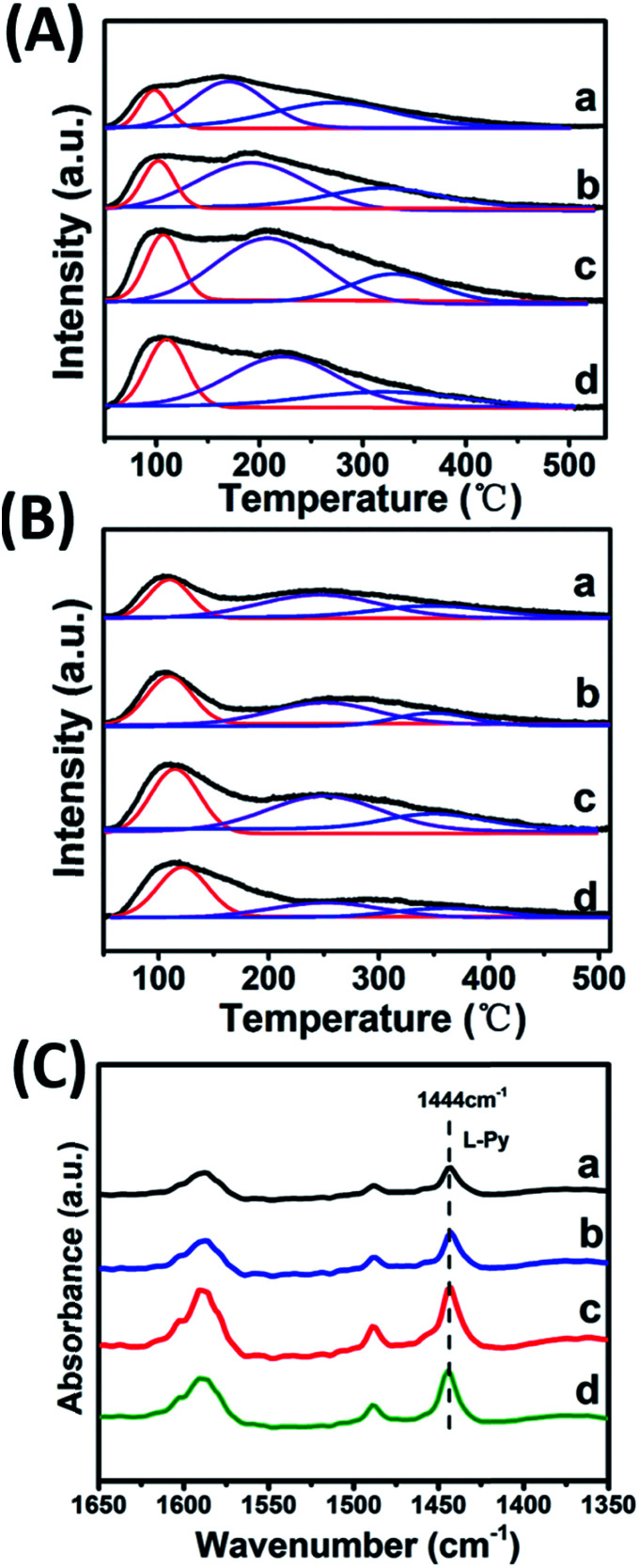
(A) CO_2_-TPD, (B) NH_3_-TPD profiles and (C) Py-IR spectra of (a) Mg_2_Zr_0.22_Al_0.78_–MMO, (b) Mg_2_Zr_0.33_Al_0.67_–MMO, (c) Mg_2_Zr_0.53_Al_0.47_–MMO, and (d) Mg_2_Zr_0.67_Al_0.33_–MMO.

CO_2_-TPD, NH_3_-TPD and IR spectroscopy of adsorbed pyridine (Py-IR) are powerful measurements to investigate the concentration and strength of basic and acid sites of catalyst,^19^ which were performed in this work (Fig. 4 and Table 2). For the CO_2_-TPD profiles, the samples show a broad CO_2_ desorption peak in the range 50–550 °C, which can be deconvoluted into three peaks with maximal temperatures (*T*_M_) in the region 120–130 °C, 170–220 °C, 300–350 °C by a Gaussian peak fitting method, corresponding to the weak, medium and strong base site, respectively. According to previous studies, the weak, medium and strong base site are derived from OH group, metal–oxygen pair and low-coordination oxygen anion, respectively.^20^ In the case of NH_3_-TPD profiles, three desorption peaks with *T*_M_ at ∼130 °C, 250 °C and 350 °C are denoted as the weak, medium and strong acid site, respectively.^21,22^ Notably, it is observed that with the increase of Zr^4+^/(Zr^4+^ + Al^3+^) ratio from 0.22 to 0.53, the amount of medium basic site or weak acid site enhances significantly, and then decreases from 0.53 to 0.67. The sample of Mg_2_Zr_0.53_Al_0.47_–MMO gives the largest amount of both medium basic site and weak acid site, showing a Zr^4+^/Al^3+^ ratio-dependent acid–base site. The increment of medium basic site can be attributed to the enrichment of Zr^4+^–O^2−^, in light of a constant Mg content (Mg^2+^–O^2−^) in all these samples. As for the increase of weak acid site, we propose that ZrO_2_ phase gives predominant contribution, since Al_2_O_3_ mainly provides medium-strong acid site.^23–25^ It should be noted that the sample of Mg_2_Zr_0.53_Al_0.47_–MMO possesses the largest specific surface area (159.45 m^2^ g^−1^), which is favorable for the exposure of medium basic or weak acid site. In addition, Py-IR measurements were performed over Mg_2_Zr_*x*_Al_1−*x*_–MMO samples to study Lewis and Brønsted acid site.^26,27^ All these samples display a characteristic band of pyridine adsorption at 1444 cm^−1^, indicating the presence of Lewis acid site originating from ZrO_2_ and Al_2_O_3_. Moreover, as the Zr/Al ratio increases, the peak intensity at 1444 cm^−1^ strengthens gradually and reaches to maximum value over the sample of Mg_2_Zr_0.53_Al_0.47_–MMO, consistent with the results of NH_3_-TPD.

**Table 2:** Concentrations of base and acid site according to CO_2_-TPD and NH_3_-TPD profiles

Sample	Base sites (mmol g^−1^)				Acid sites (mmol g^−1^)			
	*B*_TOTAL_ a	*B*_W_ b	*B*_M_ b	*B*_S_ b	*A*_TOTAL_ c	*A*_W_ d	*A*_M_ d	*A*_S_ d
Mg_2_Zr_0.22_Al_0.78_–MMO	0.311	0.045	0.151	0.115	0.277	0.074	0.143	0.060
Mg_2_Zr_0.33_Al_0.67_–MMO	0.367	0.060	0.209	0.098	0.299	0.115	0.132	0.052
Mg_2_Zr_0.53_Al_0.47_–MMO	0.543	0.103	0.315	0.125	0.436	0.160	0.224	0.052
Mg_2_Zr_0.67_Al_0.33_–MMO	0.411	0.076	0.254	0.081	0.306	0.143	0.107	0.056

a *B*_TOTAL_ is the total concentration of base sites calculated based on CO_2_-TPD.

b *B*_W_, *B*_M_, and *B*_S_ represent the concentration of weak, medium, and strong base site, respectively, which are calculated according to CO_2_-TPD and the deconvoluted TPD profiles in the temperature range 50–550 °C.

c *A*_TOTAL_ is the total concentration of acid sites calculated based on NH_3_-TPD.

d *A*_W_, *A*_M_, and *A*_S_ denote the concentration of weak, medium, and strong acid site, respectively, which are calculated according to NH_3_-TPD and the deconvoluted TPD profiles in the temperature range 50–550 °C.

### 3.3 Catalytic performance of catalysts

**Fig. 5:**
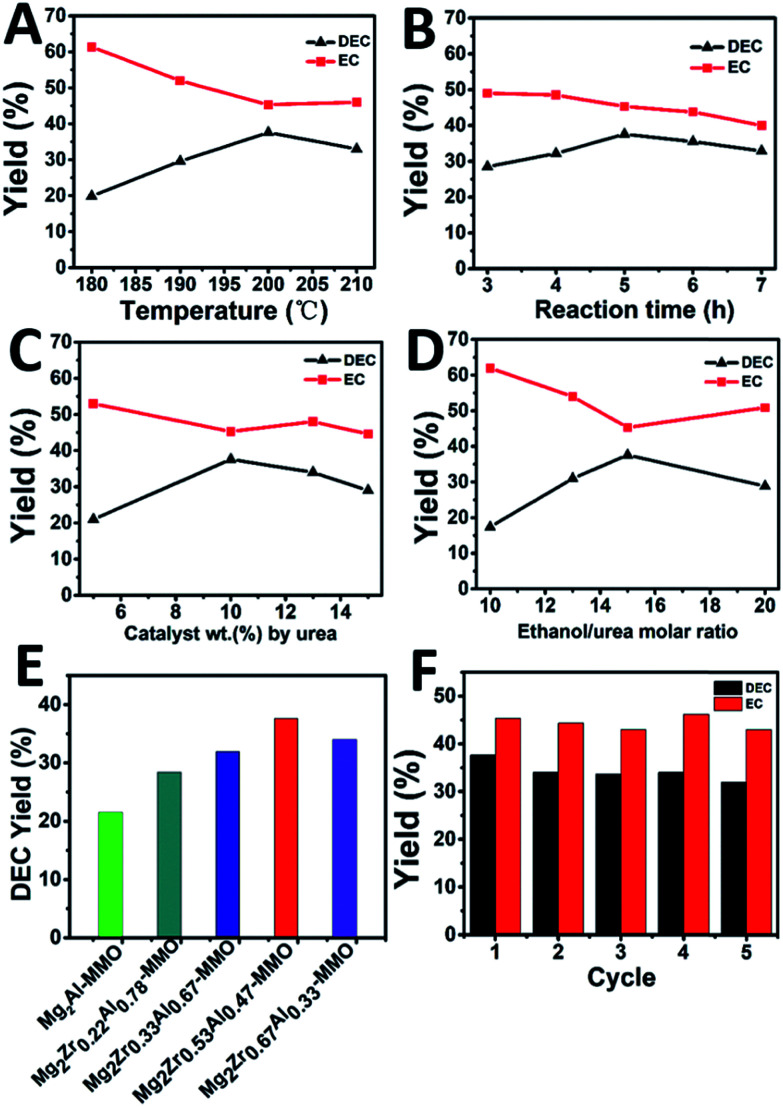
Effects of reaction conditions on the yield of DEC over Mg_2_Zr_0.53_Al_0.47_–MMO catalyst: (A) temperature, (B) reaction time, (C) the amount of catalyst and (D) ethanol/urea molar ratio as a function of DEC yield, respectively. (E) DEC yield over various catalysts under the following reaction conditions: 10 wt% catalyst (by urea), ethanol/urea molar ratio of 15, *T* = 200 °C, *t* = 5 h. (F) Catalytic performances of Mg_2_Zr_0.53_Al_0.47_–MMO in 5 consecutive recycles.

The catalytic performance of Mg_2_Zr_*x*_Al_1−*x*_–MMO catalysts toward the synthesis of DEC from ethanol and urea was studied. As shown in Fig. 5A, the yield of DEC shows an increase trend (from 19.9% to 37.6%) as the reaction temperature rises from 180 to 200 °C, followed by a decrease (from 37.6% to 33.0%) within 200–210 °C. In contrast, the intermediate product, ethyl carbamate (EC), shows an opposite change tendency, with the lowest yield at 200 °C. The reason is that a higher reaction temperature will lead to the reverse reaction from DEC to EC as well as the decomposition of EC to *N*-ethyl ethyl carbamate (side reactions).^14^ Thus, the reaction temperature is chosen as 200 °C. Fig. 5B displays the DEC synthesis as a function of reaction time, from which the largest yield of DEC is obtained at 5 h. Afterwards, the influence of catalyst weight on the catalytic behavior was also studied, and it was found that DEC yield reached the maximal value at 10% catalyst weight (Fig. 5C). Finally, as for the urea/ethanol molar ratio (Fig. 5D), the yield of DEC achieves the largest one with the ethanol/urea molar ratio of 15.

Furthermore, the catalytic performance of various Mg_2_Zr_*x*_Al_1−*x*_–MMO catalysts were evaluated under the optimum catalytic reaction (Fig. 5E and Table 3). The Mg_2_Al–MMO catalyst shows catalytic activity toward DEC synthesis, yielding 21.5% DEC. The introduction of element Zr results in a significant enhancement: the DEC yield increases gradually from 21.5% to 37.6% with the increase of Zr content, and then decreases to 34.0%; the maximum value is present in the sample of Mg_2_Zr_0.53_Al_0.47_–MMO. This is, to the best of our knowledge, among the highest level compared with previous reports.^6,11,12,14^ In addition, the reusability of Mg_2_Zr_0.53_Al_0.47_–MMO catalyst was also studied (Fig. 5F). The used catalyst was separated from the liquid product by centrifugation, washed thoroughly and dried, directly reused for the next recycle evaluation. The catalytic performance toward DEC yield shows a slight decrease after five recycles but still remains at 32.0%, indicating a satisfactory reusability.

**Table 3:** Catalytic performance of various catalysts

Sample	Product yield (%)	
	EC	DEC
Mg_2_Al–MMO	53.2	21.5
Mg_2_Zr_0.22_Al_0.78_–MMO	53.6	28.4
Mg_2_Zr_0.33_Al_0.67_–MMO	50.9	31.9
Mg_2_Zr_0.53_Al_0.47_–MMO	45.3	37.6
Mg_2_Zr_0.67_Al_0.33_–MMO	51.9	34.0

### 3.4 Studies on structure–property correlation

**Fig. 6:**
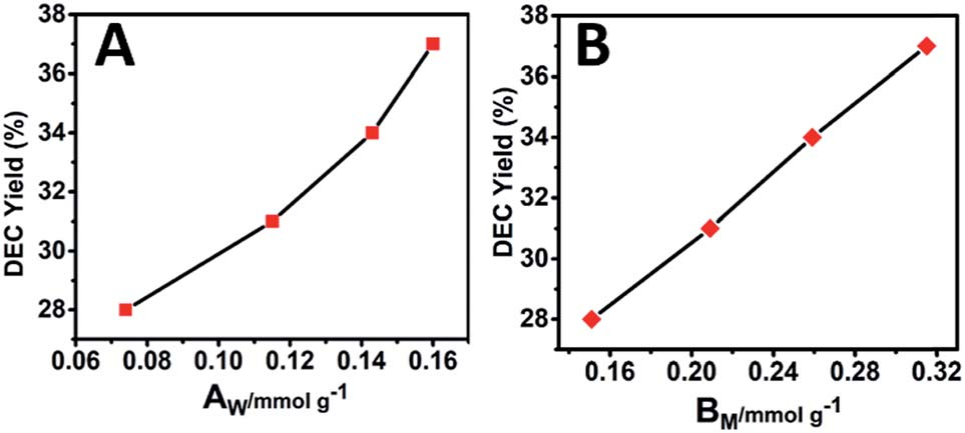
Correlations between the DEC yield and (A) the surface weak acid site (*A*_W_) or (B) surface medium strength basic site (*B*_M_) over Mg_2_Zr_*x*_Al_1−*x*_–MMO samples.

**Fig. 7:**
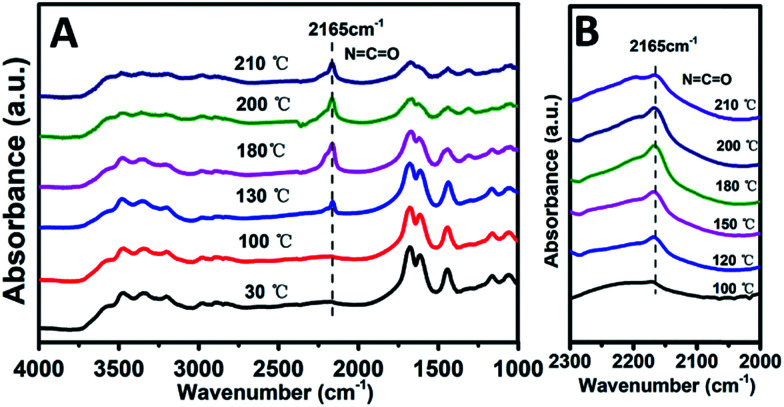
*In situ* FTIR spectra of (A) urea and (B) EC over Mg_2_Zr_0.53_Al_0.47_–MMO sample, respectively, within the temperature range 30–210 °C.

Previous studies have shown that both acidity and basicity are important to determine the catalytic activity in ethanolysis of urea.^14^ Herein, to give a deep insight into the relationship between the catalytic performance and acid–base sites of Mg_2_Zr_*x*_Al_1−*x*_–MMO samples, we correlate the DEC yield with surface acidity and basicity measured by NH_3_- and CO_2_-TPD. As shown in Fig. 6, it's interesting that the DEC yield increases along with the specific surface concentration of medium strength basicity or weak acidity, respectively, demonstrating a synergistic acid–base catalysis for the ethanolysis of urea reaction to synthesize DEC. To further investigate the cooperation mechanism between medium strength basicity and weak acidity and their respective contribution, *in situ* FTIR of reactants on the catalysts was conducted. The ethanolysis of urea includes two consecutive steps: the formation of intermediate ethyl carbamate (EC) from urea and ethanol, followed by the subsequent conversion to DEC. The second step (reaction of EC with ethanol), is regarded as the rate-limiting step for its unfavorable thermodynamics.^2^ Thus, we carried out the adsorption measurements of urea, EC and DEC over Mg_2_Zr_0.53_Al_0.47_–MMO and control sample (Mg_2_Al–MMO), respectively. Fig. 7A shows the urea adsorption over Mg_2_Zr_0.53_Al_0.47_–MMO within the temperature range 30–210 °C. At room temperature, the characteristic bands of urea are observed: bands at 3436 cm^−1^ and 3345 cm^−1^ are due to N–H stretching vibrations; bands at 3213 cm^−1^ is attributed to the N–H stretching vibration with intermolecular association; bands at 1681 cm^−1^ and 1161 cm^−1^ are assigned to C=O and C–N stretching vibrations. A new band at 2165 cm^−1^ appears at 130 °C with gradually enhanced intensity until 200 °C, accompanied with decreased bands of urea. This band is assigned to the formation of metal isocyanato group: O=C=N bound to the metal oxide surface (O=C=N–M asymmetric stretching, M represent Al^3+^, Zr^4+^),^15^ as a result of the adsorption of urea onto Lewis acid sites (ZrO_2_ and Al_2_O_3_ in Mg_2_Zr_0.53_Al_0.47_–MMO catalyst).

Furthermore, the adsorption of EC on the catalysts was studied. As shown in Fig. 7B, a band at 2165 cm^−1^ corresponding to the stretching vibration of O=C=N bound to the metal oxide surface (O=C=N–M asymmetric stretching, M represent Al^3+^, Zr^4+^) is observed, whose intensity increases as the temperature rises from 100 to 200 °C, indicating that EC is effectively activated. Compared with EC adsorption on Mg_2_Al–MMO catalyst (Fig. S4:† O=C=N–M stretching at 2160 cm^−1^, adsorption temperature at 180 °C), a higher frequency of 2165 cm^−1^ and a lower temperature of 100 °C are observed for Mg_2_Zr_0.53_Al_0.47_–MMO catalyst. This lower temperature implies the activation of EC on Mg_2_Zr_0.53_Al_0.47_–MMO is more favorable than that on Mg_2_Al–MMO catalyst. A higher frequency indicates a stronger O=C=N bond but a weaker interaction between O and acid site in Mg_2_Zr_0.53_Al_0.47_–MMO.^2^ As discussed above, Mg_2_Zr_0.53_Al_0.47_–MMO catalyst possesses mainly weak acid site deprived from ZrO_2_ while Mg_2_Al–MMO offers medium–strong acid site (Fig. S2†) provided by Al_2_O_3_. In other words, weak Lewis acid site serves as main active site to activate EC over Mg_2_Zr_0.53_Al_0.47_–MMO while medium–strong Lewis acid site accounts for the activated adsorption of EC over Mg_2_Al–MMO. This is in accordance with the correlation between DEC yield and surface weak acidity measured by NH_3_-TPD (Fig. 4). The activation of EC by weak Lewis acid site over Mg_2_Zr_0.53_Al_0.47_–MMO catalyst is more efficient than by the medium–strong Lewis acid site over Mg_2_Al–MMO sample, which promotes the conversion of EC to DEC (the rate-limiting step) with a higher yield of DEC.

**Fig. 8:**
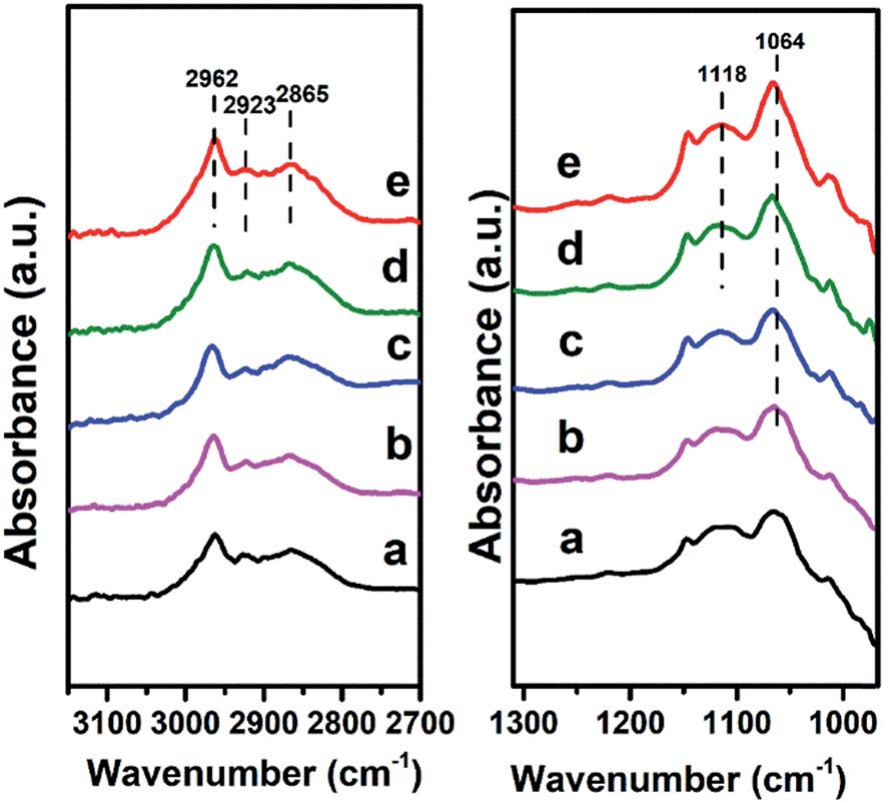
*In situ* FTIR spectra evolution of ethanol over Mg_2_Zr_0.53_Al_0.47_–MMO at: (a) 100 °C, (b) 120 °C, (c) 150 °C, (d) 180 °C, (e) 200 °C, respectively.

Finally, we investigated the adsorption behavior of ethanol over Mg_2_Zr_0.53_Al_0.47_–MMO at various temperatures (Fig. 8). In the CH_2_/CH_3_ stretching region, two peaks at 2962 and 2923 cm^−1^ are attributed to CH_3_ stretching and the peak at 2865 cm^−1^ is due to CH_2_ stretching in ethoxide.^28^ In C–C–O stretching regions, the band at 1064 cm^−1^ and 1118 cm^−1^ are assigned to adsorbed ethoxide species^28^ over the catalyst, which indicates that ethanol is effectively activated over Mg_2_Zr_0.53_Al_0.47_–MMO through the abstraction of H^*δ*+^ by Lewis basic site. Additionally, the peaks intensity of ethoxide species increases gradually when the temperature rises from 100 °C to 200 °C, indicating an enhanced activation of ethanol over Mg_2_Zr_0.53_Al_0.47_–MMO at a higher temperature. In comparison, Mg_2_Al–MMO sample displays two peaks at 1102 cm^−1^ and 1165 cm^−1^ (Fig. S5†) in C–C–O stretching region, corresponding to the adsorbed ethoxide species.^29^ The large difference in band position suggests that the basic sites provided by the two catalysts are different for the activation of ethanol: Zr^4+^–O^2−^ as main basic site is responsible for the activated adsorption of ethanol (ethoxide species) over Mg_2_Zr_0.53_Al_0.47_–MMO while Mg^2+^–O^2−^ serves as predominant active center in Mg_2_Al–MMO sample. Previous study reported that weak basic site can not activate ethanol in this catalytic system.^4^ Thus, based on the combination of *in situ* FTIR spectra and CO_2_-TPD results, it is proposed that the presence of medium strength Lewis basic sites (Zr^4+^–O^2−^, Mg^2+^–O^2−^) in Mg_2_Zr_0.53_Al_0.47_–MMO facilitates the activated adsorption of ethanol to produce ethoxide which is involved in the following reaction.

## 4. Conclusions

In summary, acid–base Mg/Zr/Al mixed metal oxides (denoted as Mg_2_Zr_*x*_Al_1−*x*_–MMO) were successfully synthesized *via* the calcination transformation of Mg_2_Zr_*x*_Al_1−*x*_–LDH precursors. The obtained Mg_2_Zr_*x*_Al_1−*x*_–MMO catalysts are evaluated by the ethanolysis of urea reaction to produce DEC, and the sample of Mg_2_Zr_0.53_Al_0.47_–MMO demonstrates the largest DEC yield of 36.7%. Studies on structure–activity correlation reveal a cooperative catalysis between medium strength base site and weak acid site of Mg_2_Zr_0.53_Al_0.47_–MMO in this reaction. Moreover, CO_2_- and NH_3_-TPD study as well as the *in situ* FTIR measurements verify that the medium strength Lewis base sites (Zr^4+^–O^2−^, Mg^2+^–O^2−^) serve as active center to activate ethanol to produce ethoxide species; while weak Lewis acid sites (mostly ZrO_2_) act as active site toward EC activation to produce O=C=N–M. Therefore, a synergistic acid–base catalysis is demonstrated in the Mg_2_Zr_0.53_Al_0.47_–MMO catalyst, accounting for its higher DEC yield toward ethanolysis of urea reaction.

## Conflicts of interest

There are no conflicts to declare.

## Supplementary Material

RA-008-C7RA13629C-s001
