# Supplementary material for: Acid–base sites synergistic catalysis over Mg–Zr–Al mixed metal oxide toward synthesis of diethyl carbonate
Source: RSC Adv. 2018 Jan 26;8(9):4695–702. doi: 10.1039/c7ra13629c (PMC9077746; doi:10.1039/c7ra13629c)
Supplement: RA-008-C7RA13629C-s001 [file RA-008-C7RA13629C-s001.pdf]

Electronic Supplementary Material (ESI) for RSC Advances.
This journal is © The Royal Society of Chemistry 2018

Supporting Information for

Acid-Base Sites Synergistic Catalysis over Mg-Zr-Al Mixed Metal Oxide toward Synthesis of Diethyl Carbonate

Tingting Yan, Weihan Bing, Ming Xu,Yinwen Li, Yusen Yang, Guoqing Cui, Lan Yang* and Min Wei*

State Key Laboratory of Chemical Resource Engineering, Beijing Advanced Innovation Center for Soft Matter Science and Engineering, Beijing University of Chemical Technology, Beijing 100029, P. R. China

CORRESPONDING AUTHOR FOOTNOTE

∗ Corresponding authors. Phone: +86-10-64412131. Fax: +86-10-64425385. E-mail: yanglan@mail.buct.edu.cn; weimin@mail.buct.edu.cn.

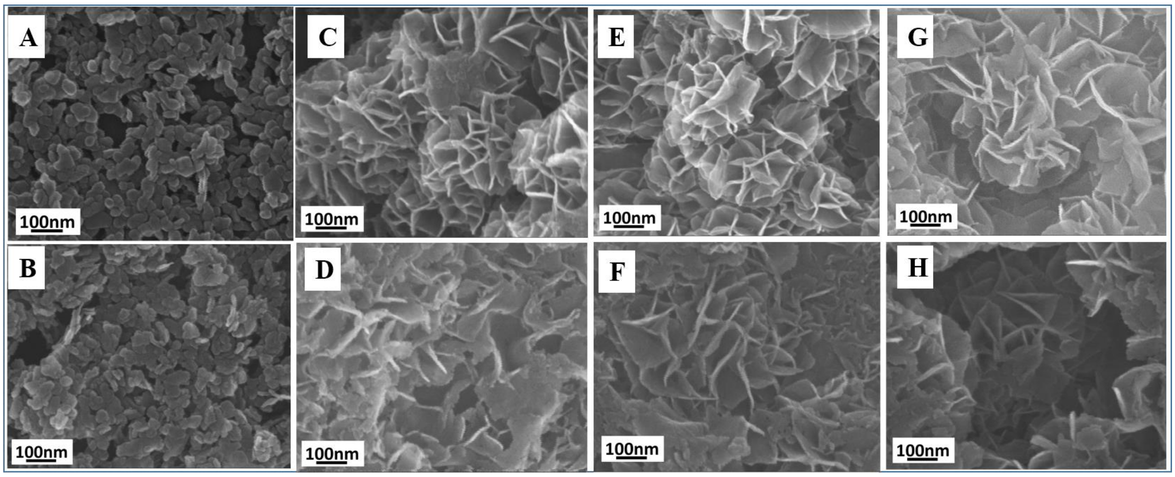

Figure S1. SEM images of (A) Mg_2Al-LDH, (B) Mg_2Al-MMO, (C) $Mg_2Zr_{0.22}Al_{0.78}$-LDH, (D) $Mg_2Zr_{0.22}Al_{0.78}$-MMO, (E) $Mg_2Zr_{0.33}Al_{0.67}$-LDH, (F) $Mg_2Zr_{0.33}Al_{0.67}$-MMO, (G) $Mg_2Zr0_{.67}Al_{0.33}$-LDH, (H) $Mg_2Zr0_{.67}Al_{0.33}$-MMO.

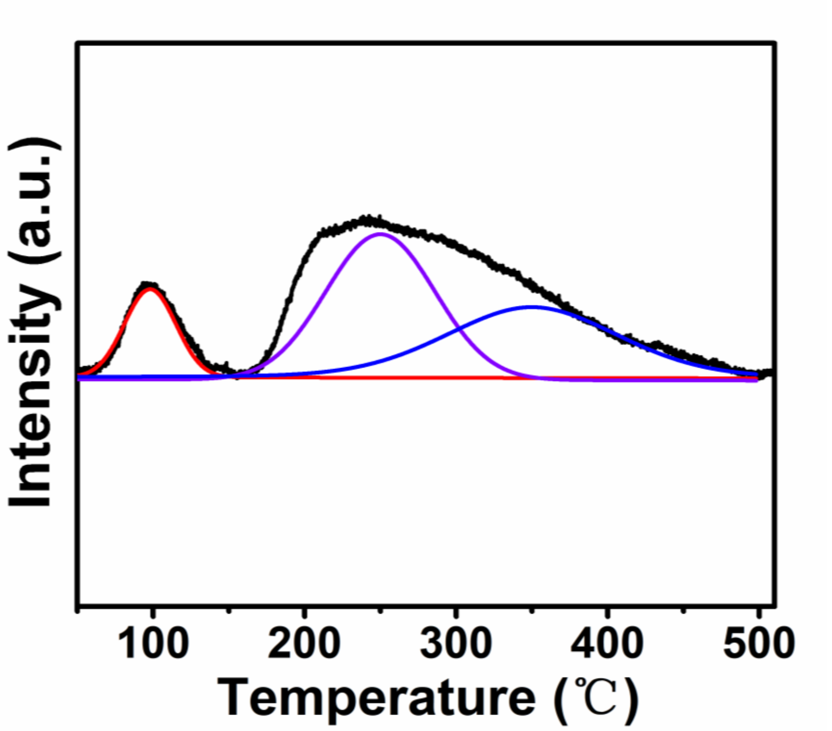

Figure S2. NH_3-TPD profiles of Mg_2Al-MMO sample.

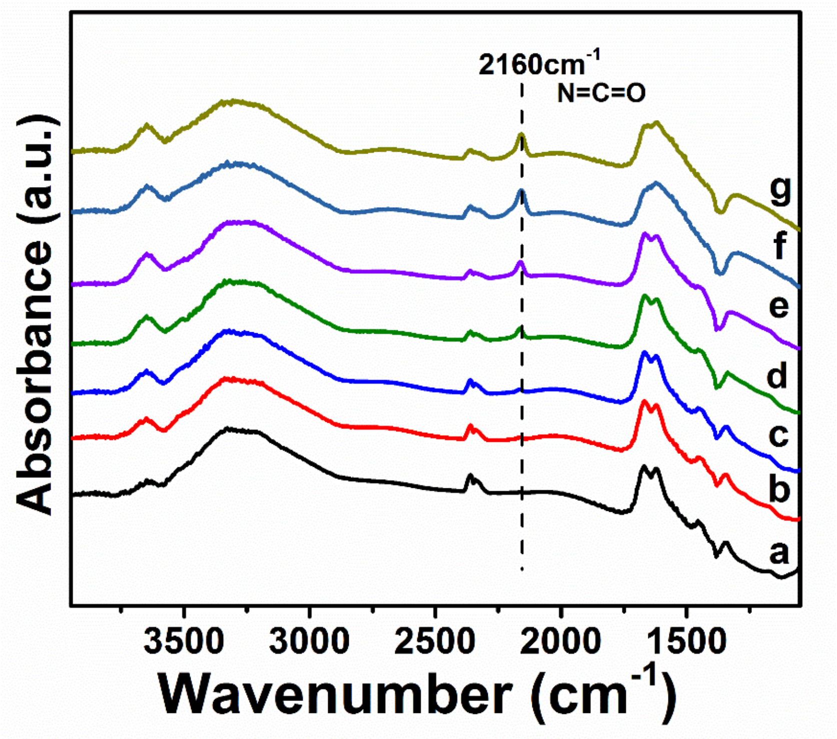

Figure S3. *In situ* FTIR spectra evolution of urea over Mg_2Al-MMO sample at: (a) 30 °C, (b) 100 °C, (c) 130 °C, (d) 150 °C, (e) 180 °C, (f) 200 °C, (g) 210 °C, respectively.

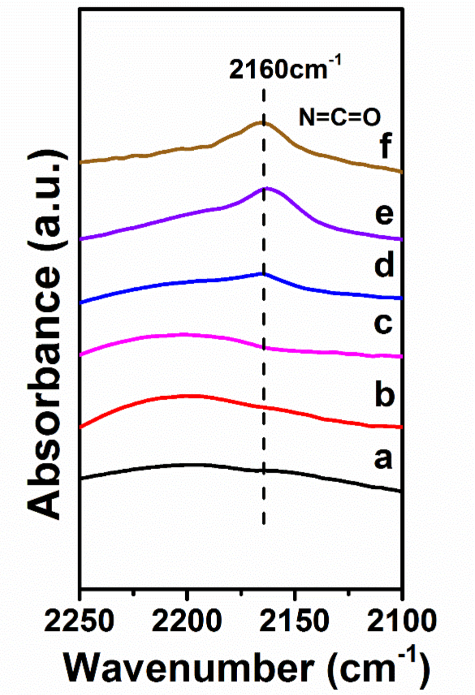

Figure S4. *In situ* FTIR spectra evolution of EC over Mg_2Al-MMO sample at: (a) 100° C, (b) 120 °C, (c) 150 °C, (d) 180 °C, (e) 200 °C, (f) 210 °C, respectively.

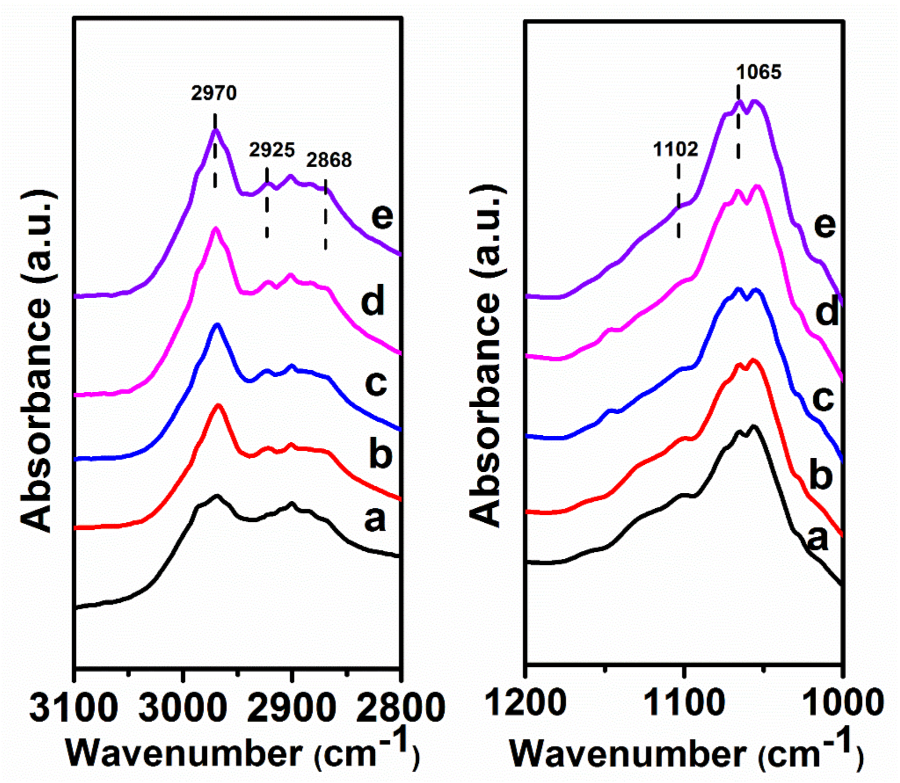

Figure S5. *In situ* FTIR spectra evolution of ethanol over Mg_2Al-MMO sample at: (a) 100 °C, (b) 120 °C, (c) 150 °C, (d) 180 °C, (e) 200 °C, respectively.
